# A needle in the haystack – the dire straits of needle exchange in Hungary

**DOI:** 10.1186/s12889-016-2842-2

**Published:** 2016-02-16

**Authors:** V. Anna Gyarmathy, Róbert Csák, Katalin Bálint, Eszter Bene, András Ernő Varga, Mónika Varga, Nóra Csiszér, István Vingender, József Rácz

**Affiliations:** Johns Hopkins Bloomberg School of Public Health, Baltimore, MD USA; Semmelweis University, Faculty of Health Sciences, Budapest, Hungary; Blue Point Drug Counselling and Outpatient Centre, Budapest, Hungary; Eötvös Loránd University, Institute of Psychology, Budapest, Hungary; Drug Prevention Foundation, Budapest, Hungary

**Keywords:** People who inject drugs, Harm reduction, Needle exchange, Drug policy, Hungary

## Abstract

**Background:**

The two largest needle exchange programs (NEPs) in Hungary were forced to close down in the second half of 2014 due to extreme political attacks and related lack of government funding. The closures occurred against a background of rapid expansion in Hungary of injectable new psychoactive substances, which are associated with very frequent injecting episodes and syringe sharing. The aim of our analysis was to predict how the overall Hungarian NEP syringe supply was affected by the closures.

**Methods:**

We analyzed all registry data from all NEPs in Hungary for all years of standardized NEP data collection protocols currently in use (2008–2014) concerning 22 949 client enrollments, 9 211 new clients, 228 167 client contacts, 3 160 560 distributed syringes, and 2 077 676 collected syringes.

**Results:**

We found that while the combined share of the two now closed NEPs decreased over time, even in their partial year 2014 they still distributed and collected about half of all syringes, and attended to over half of all clients and client contacts in Hungary. The number of distributed syringes per PWID (WHO minimum target = 100) was 81 in 2014 in Hungary, but 39 without the two now closed NEPs.

**Conclusions:**

There is a high probability that the combination of decreased NEP coverage and the increased injection risk of new psychoactive substances may lead in Hungary to a public health disaster similar to the HIV outbreaks in Romania and Greece. This can be avoided only by an immediate change in the attitude of the Hungarian government towards harm reduction.

## Background

Injecting drug use is a major risk factor in the transmission of HIV and Hepatitis C virus (HCV) [1, 2]. There is substantial evidence to support the effectiveness of harm reduction interventions such as needle exchange programs (NEP) in reducing HIV and HCV transmission among people who inject drugs (PWIDs) [2–7], although the link between HCV transmission and NEP alone is unclear [3, 7]. A large body of research has emphasized the importance of complex interventions, such as the integration of NEPs, drug treatment, anti-viral treatment and other harm reduction interventions as effective strategies in combating the HIV and HCV epidemic among PWIDs [5, 6, 8–10]. Therefore, we can conclude that NEPs are key elements of effective HIV and HCV prevention strategies [11], even regardless of the income level of the country where they are located (high income vs. middle income vs. low income countries), indicating that it is the effectiveness of NEPs to reach PWIDs – also referred to as “coverage” – rather than the economic background *per se* that really matters [12].

In their publication updated in 2012 [13], the World Health Organization (WHO) underlines the importance of NEPs, among other sources of sterile needles and syringes, not only in their role of preventing drug related infectious diseases, but also as important contacts with and potential entry points to health services. The number of distributed syringes per PWID per year is a WHO indicator (reference number: NSP.C.1c) to measure NEP coverage [13]. WHO sets the minimum target in this indicator at 100 (low coverage) and the desirable target at 200 (high coverage) for HIV prevention, noting that levels required for HCV prevention are likely to be much higher than these values [13].

After HIV was discovered in 1981 as the cause of AIDS, guidelines and recommendations were developed to curb the HIV epidemic among PWIDs as well [14]. However, HIV is still very prevalent in many PWID populations around the world [15]. One reason for this is the limited resources available in certain countries for harm reduction among PWIDs, but probably the main reason is a lack of supporting policies or political will linked to the stigmatization of drug users and drug use [14]: while higher income countries have more developed and better implemented harm reduction programs (including NEPs) among PWIDs, this is not the case in much of the middle or low income countries. For example, the sustained extremely low HIV transmission rates among PWIDs in Australia have been attributed to early and sustained harm reduction responses (such as NEPs) backed by strong drug policy and political support [16]. On the other hand, the United States has the lowest level of NEP availability in the developed world [17], which probably has a lot to do with a federal ban on funding NEPs [18].

The first NEP in Hungary opened in 1994 under the auspices of the Drug Prevention Foundation (DPA), using a loophole in Hungarian legislation. In the early 2000s, NEPs already played an important role: 40 % of street recruited PWIDs reported that they obtained all their syringes from the NEP [19], and the Hungarian National Focal Point (HNFP) estimated that about 49 % of PWIDs were reached by NEP services [20]. By the end of 2014, there were 31 NEPs in the country – the largest (in terms of number of clients, contact, and syringes distributed) being Blue Point and the second largest being DPA. At this point, there is still a lack of either legal restriction or legal permission of needle exchange operations in Hungary. However, DPA and Blue Point NEPs closed in Budapest at the end of 2014 due to political attacks and a related considerable reduction in government funding for harm reduction activities and programs [21]. Much of these political attacks had to do with blames against the NEPs of “distributing syringes” instead of “exchanging syringes”, due to their so-called “one-for-one plus” exchange policy, where NEPs exchange syringes but also give additional syringes in addition to the number that was exchanged [22], and with the gentrification of the districts where these needle exchanges were operating. A corroborating body of research, however, shows that actually “distributing syringes” (meaning: giving out the amount of syringes based upon need as opposed to how many syringes were turned in by the clients) was associated with lower odds of syringe re-use – although not with receptive or distributive syringe sharing – when compared to “one-for-one” or “one-for-one plus” syringe exchange policies [22–25]. In addition, increased access to sterile syringes – not surprisingly – lead to a decrease in both distributive and receptive syringe sharing, and consequently to declining HIV incidence in a prospective cohort study among over a thousand PWIDs in Vancouver, Canada [26].

Given that these were the two largest NEPs not only in Budapest, but also in Hungary, the closures may have a largely detrimental effect on harm reduction efforts in the country. The goal of this analysis was to predict how the overall Hungarian NEP syringe supply was affected by the closures, by calculating the combined share of Blue Pont and Drug Prevention Foundation (DPA) NEPs across five turnover data types using all turnover data from all NEPs in Hungary during the years between 2008 and 2014, and by calculating how the number of distributed syringes per PWID per year decreased as a result of the closures.

## Methods

### Data sources

Collection of standardized NEP registry data (also referred to as “NEP client turnover data”) started in 2003 in Hungary. In 2008 the protocol was slightly changed to include data for new clients as well. In this study, we analyze all client turnover data for altogether 22 949 client enrollments, 9 211 new clients, 228 167 client contacts, 3 160 560 distributed syringes, and 2 077 676 collected syringes from all NEPs in Hungary between 2008 and 2014, that is, for all years of standardized NEP data collection protocols that are currently in use, until the year when Blue Point and DPA were closed (year 2014). Since Blue Point NEP was closed down in August 2014 and DPA was closed down in November of 2014, they contributed only seven and ten full months, respectively, to year 2014, while all other NEPs contributed 12 months of data. During this period, the number of NEPs gradually increased from 18 in 2008 to 31 at the end of 2014.

As part of routine data collection, Hungarian NEPs record and then aggregate at the end of each year five types of turnover data: number of clients, number of new clients, number of client contacts, number of distributed syringes, and number of collected syringes. The “number of clients” variable refers to client enrollments, meaning it is the number of unique clients who had at least one visit at a given NEP during a given year. Double entries for the same client are deleted at the level of service providers, but not at national level. The “number of new clients” variable – a subset of the “number of clients” variable – denotes the number of individuals during a given year who register for the first time into the database of a particular NEP. One client contact means one visit by a client in the NEP; therefore, the “number of client contacts” variable describes all visits by all clients at a particular needle exchange during a given year. The “number of distributed syringes” is the number of all syringes handed out to clients by a given NEP in a given year. The “number of collected syringes” is the sum of the number of used syringes that were returned by clients to the NEP and the number of syringes that were collected by NEP staff (e.g. on the streets or in parks).

All Hungarian NEPs are obliged to report their respective aggregate client turnover data to the HNFP, who is then obliged to report a summary of the aggregate data to the European Monitoring Centre for Drugs and Drug Addiction (EMCDDA) in form of a national report. The HNFP usually releases the official NEP data in the middle of the year for the previous year (e.g. data were released on 24 June 2015 for the year 2014). For this analysis, we used a combination of individual aggregate NEP data and aggregate summary NEP data, as follows: annual aggregate data referring to Blue Point NEP and the NEP operated by DPA were provided directly to the authors by the respective NEPs. Annual aggregate summary data concerning all other NEPs in Hungary, stratified by two geographical locations (in Budapest vs. outside of Budapest), were provided by the HNFP. The collection of primary data that constitutes the basis for this secondary analysis was ethically approved by the Supervisory Boards of the respective NEPs.

### Data management and analysis

We calculated the following two indicators for each year: 1. the combined share of the two needle exchange programs operated by Blue Point and DPA (this indicator was calculated for all five turnover data types), 2. the number of distributed syringes per PWID per year.

The combined share of the two needle exchange programs operated by Blue Point and DPA was calculated, for each five turnover data types, as: *(the total number of [turnover data type] for Blue Point + the total number of [turnover data type] for DPA) / the total number of [turnover data type] for all NEPs in Hungary*.

The original equation to calculate the number of distributed syringes per PWID per year [13] also includes the number of syringes sold to PWID in pharmacies and other outlets. Pharmacy sales data, however, are not recorded in Hungary – similarly to most countries [13]. Therefore, we calculated this indicator as: *the total number of syringes distributed by all NEPs in Hungary in a given year / the estimated number of PWIDs in Hungary.* The only available estimation of the size of the PWID population in Hungary was calculated in 2010 [27] using the capture-recapture method, and it set the figure at 5 699 persons.

## Results

**Fig. 1:**
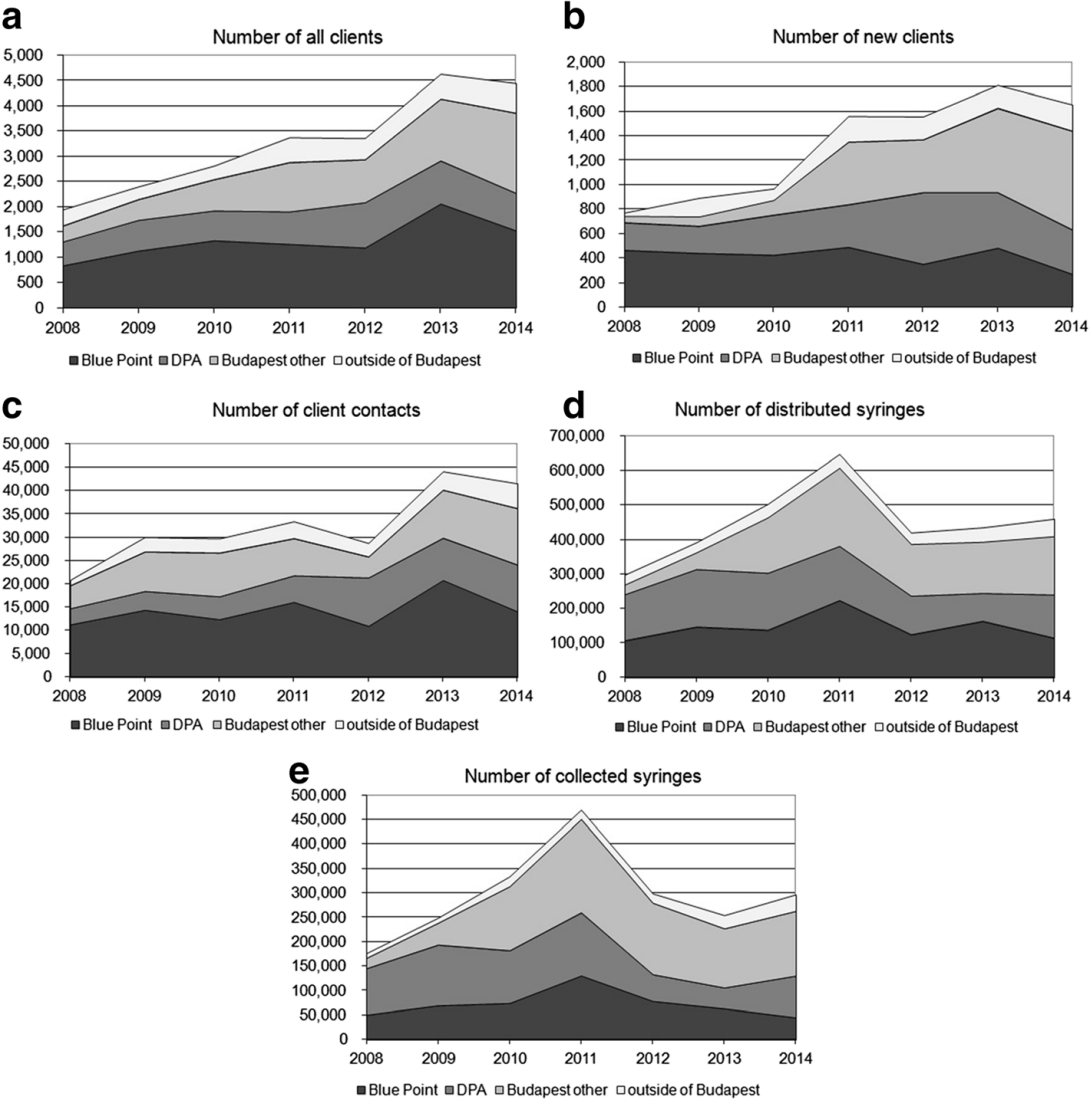
Client turnover data for all clients in all NEPs in Hungary between 2008 and 2014, by NEP (Note: NEPs in Budapest other than Blue Point and DPA are aggregated as one group, and NEPs outside of Budapest are aggregated as one group). **a** Number of all clients. **b** Number of new clients. **c** Number of client contacts. **d** Number of number of distributed syringes. **e** Number of number of collected syringes.

During the seven years of the study, the number of both all and new clients, and the number of client contacts showed an overall increase in Hungary (Fig 1a, b and c). By contrast, the number of distributed and collected syringes increased until 2011, then in 2012 dropped sharply and stayed at about the same level for three years (Fig. 1d and e). About 90 % of all client indicators were recorded in Budapest and about 10 % outside of the city.

**Fig. 2:**
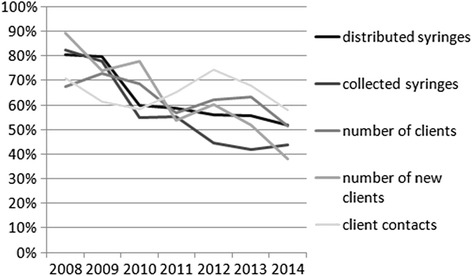
Blue Point and DPA NEPs’ combined share of all clients, new clients, client contacts, distributed syringes, and collected syringes of all clients and all NEPs in Hungary between 2008 and 2014

**Fig. 3:**
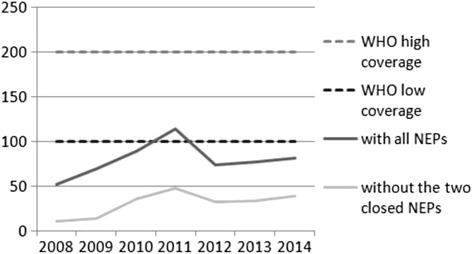
The number of distributed syringes per PWID per year in Hungary between 2008 and 2014

Blue Point's and DPA’s combined share of distributed and collected syringes, and number of both all and new clients declined over time, but their combined share of client contacts showed an increasing trend (Fig. 2). In their last full year of operation, in 2013, Blue Point and DPA together distributed and collected about half of all syringes, and attended to about two thirds of all clients and client contacts in Hungary. In the year of their closure, in 2014, their share was about half for all client indicators. The number of distributed syringes per PWID per year increased constantly until 2011 (where it peaked at 114), then by 2012 it decreased sharply and stayed at a level of about 80 for three years (81 in 2014) – when the NEPs operated by Blue Point and DPA are excluded, this number drops to 39 in 2014 (Fig. 3).

## Discussion

The two largest NEPs in Hungary were forced to close down after the second third of 2014 due to extreme political pressure and related lack of government funding. The aim of our analysis was to assess how the overall Hungarian NEP syringe supply was affected by the closures, by assessing all turnover data from all NEPs in Hungary between 2008 and 2014. First, we would like to explain and put into context our findings, and then elaborate on the context and what the closure of these NEPs means not only for the functioning of the Hungarian harm reduction services but also for the epidemiological situation of the country.

There was an increase in all turnover data between 2008 and 2011, for which there are several reasons. First, an increase in the number of both clients and contacts, and distributed and returned syringes may be the reflection of an increase in the access of PWIDs to NEPs due to an increase in NEP awareness and/or reduction in the stigma associated with accessing harm reduction services. Second, it may reflect an increasing demand for injecting equipment linked to the increasing prevalence of use of injectable new psychoactive substances (NPS), especially cathinones, which are associated with higher injecting frequencies than “traditional” drugs, such as heroin or amphetamine [28].

After the Fidesz - Hungarian Civic Alliance entered into power in 2010 [29], there was an immediate attack on Hungary’s drug policy and all harm reduction activities [21]. This resulted in a drastic cut in 2011 in needle exchange funding for the year 2012 and thereafter. The cuts in funding are reflected in our data: the number of both distributed and collected syringes plummeted in 2012, while the number of all and new clients, and client contact kept increasing, indicating the presence of demand for injecting equipment.

While by 2011 Hungary slightly exceeded the minimal WHO target of 100, between 2012 and 2014 the indicator fell back to a pre-2010 level of about 80 (about two thirds of what it was a year before). Moreover, when the two now closed largest NEPs are excluded, the indicator vacillates in the miserly thirties after 2011. Since there is no indication whatsoever that any of Blue Point and DPA NEP funding will be redistributed to other NEPs in Budapest or Hungary, or that there will be an increase in NEP funding for the NEPs that are still open, we expect the number of syringes distributed per PWID per year in Hungary to stay in the low thirties in the upcoming years as well. In addition, some populations, such as the segregated, low SES and mainly Roma populations that Blue Point NEP was serving, will be largely unserved [21]. Therefore, the NEP coverage in Hungary will not be sufficient to effectively prevent an HIV epidemic.

Due to a considerable decrease in funding, NEPs had to institute caps on distributed syringes: for example, until May 2012, clients at Blue Point were allowed to exchange an unlimited number of used needles, whereas afterwards the maximum number of exchangeable syringes was reduced to between 2 and 5 per client per visit (depending on actual availability of funding). Similar restrictions were instituted at DPA as early as 2011. Due to the restrictions, we estimate that fewer of the distributed syringes were returned to the NEPs and more of them stayed in circulation (most probably for reuse) after being used. As a matter of fact, clients at Blue Point NEP reported to staff that they kept the used syringes for later reuse, for as many as 6–8 times, due to a lack of access to sterile syringes.

The question arises: why don’t PWIDs just go to the pharmacy to buy syringes? The answer is multifaceted, but has one interesting element. PWIDs in Hungary traditionally use one-piece diabetic syringes [30], which up till the recent past were easily available in any pharmacy. Due to the wide adoption of new technology, however, about 90 % of diabetic patients in Europe (in Hungary as well) use insulin pens instead of diabetic syringes [31]. This means that those syringes that PWIDs use to inject drugs can be purchased only at selected pharmacies and only in small quantities, and generally only two-piece syringes are available now in pharmacies (Gyarmathy, personal communication). Not only are the needles of two-piece syringes wider and thereby more uncomfortable to inject with (causing more vein damage and epidermal infections), but their use has also been found to be associated with a higher risk of both HIV and HCV transmission [28, 32–34].

Some limitations are noteworthy. As we noted in the methods, duplicate client data are deleted at the service provider level, but not at national level. Therefore, some clients may be registered with more than one NEP, and thereby counted two or more times. Staff at Blue Point, however, regularly walk around the city and pass by other NEPs, and they reported very little overlap among the clientele of different NEPs. Furthermore, the fact that almost 90 % of Blue Point clients live in the 8^th^ district, which is the district where Blue Point is located, and only 1 % live in the 13^th^ district, which is the district where DPA is located [35], suggests that the overwhelming majority of clients go to the NEP that is located close to where they live and only a few percent may be double counted by also visiting NEPs outside their living area. Another limitation is that the increase in the number of client contacts and all and new clients may be an artefact due to a limit in the number of needles clients could take as a result of limited funding: clients compensated for the lower number of syringes they could take with more frequent visits to the NEPs and more PWID had to visit the NEPs as sending one’s used syringes with someone else was not a viable option anymore (since one client could exchange only a limited number of syringes). While some of the increase may be explained with this, much of it is part of a trend: a continuation of a decade long upward trend in client and contact numbers. Furthermore, we had only one estimate (from 2010) of the number of PWIDs in Hungary, and we used this for all years to calculate the number of syringes per PWID per year. The only available estimation does not reflect the changes occurred during the recent years, namely that due to the emergence of NPS – much of which require injecting – there may have been an increase in the number of PWIDs in Hungary. It is likely that the actual population before 2010 could be somewhat smaller and after 2010 it could be somewhat larger than the estimation we used; therefore, the number of syringes per PWID per year indicator that we have calculated may be somewhat imprecise and probably overestimated. Since no confidence limits were published along with the estimate of PWIDs, we are unable to report estimates of uncertainty for the number of syringes distributed per person per year. In addition, since pharmacy syringe distribution data are unavailable, coverage estimates may be somewhat underestimated. With the closure of the two largest NEPs in Hungary, about two thirds of PWIDs who are NEP clients may now be unserved or underserved, and it is likely that the number of distributed sterile one-piece syringes will have decreased to about half. As a result, the number of syringes distributed per PWID per year will probably fall to just over a third of the minimum level recommended by WHO.

Given the fact that the latest data for this analysis are from 2014, and data for the year 2015 are available only in mid-2016, we were unable to assess the actual NEP availability for the year 2015. During 2015, no qualitative or quantitative studies in Hungary assessed the effect of the 2014 NEP closures. A documentary film that was produced in the middle of 2015, however, supports our analysis findings [36]. While there is a new foundation with a handful of people distributing syringes on the street on certain days, their provision capacity is a negligible fraction of what the now two closed NEPs provided. Drug users interviewed in this film complain about the unavailability of syringes and that as a result they are resorted to using one syringe for up to one month, picking up used syringes from the street to reuse, and to sharing the same syringe multiple times. Another problem that drug users mentioned was the lack support other than syringe provision and lack of service access that were available at the NEPs that are now closed. This, clearly, unerlines our findings and points to a dramatic increase in injecting risk.

The changes that we describe in this analysis occurred against a background of rapid expansion of NPS (especially synthetic injectable cathinones), which are associated with a higher number of injecting episodes, a drastic increase in daily injecting, and an increase in syringe sharing (resulting from a combination of more injecting and fewer available sterile syringes) [21, 28], and a reduction in the availability of one-piece diabetic syringes in pharmacies due to the wide dissemination of diabetic pens. To put this into context: the current level in Hungary of the number of syringes distributed per PWID per year is even below the levels that existed in Romania before the HIV outbreak among PWIDs started in 2011 (55 in Romania in 2010 vs. in the 30s in Hungary now) [13]. In addition, a substantial increase in drug injecting and syringe sharing took place both in Greece and in Romania just a few years before the HIV epidemic among PWIDs in 2011 and 2012.

## Conclusions

There is a high probability that the combination of decreased access to sterile one-piece syringes (and the resulting move to injecting with two-piece syringes), and NPS that are associated with considerably more frequent injecting episodes may lead in Hungary to a public health disaster similar to the HIV outbreaks in Romania and Greece. As a matter of fact, between 2011 and 2014 (during the time when the number of syringes per PWID per year dropped from 114 to 39) the prevalence of HCV among PWIDs in Hungary doubled (from 24 % to 49 % in the country and from 34 % to 60 % in Budapest, respectively), indicating that the epidemiological predisposition for a drug injecting related infectious disease epidemic is present - putting an end to an era of near-zero HIV prevalence among PWIDs [37]. This can be avoided only by a change in the attitude of the Hungarian government towards harm reduction and a related scaling up of NEP services.
